# Urinary Exosomal MiRNA-4534 as a Novel Diagnostic Biomarker for Diabetic Kidney Disease

**DOI:** 10.3389/fendo.2020.00590

**Published:** 2020-08-28

**Authors:** Yanyan Zhao, Ao Shen, Feng Guo, Yi Song, Na Jing, Xiaoxu Ding, Mengxing Pan, Haohao Zhang, Jiao Wang, Lina Wu, Xiaojun Ma, Liang Feng, Guijun Qin

**Affiliations:** ^1^The First Affiliated Hospital of Zhengzhou University, Zhengzhou, China; ^2^School of Traditional Chinese Pharmacy, China Pharmaceutical University, Nanjing, China

**Keywords:** urinary exosomes, MiRNA, diabetic kidney disease, biomarker, diagnostic value

## Abstract

Urinary exosomal miRNAs can reflect the physiological and possibly pathophysiological state of cells lining the kidney and participate in the regulation of transcription and translation of proteins, which are playing an important role in the pathogenesis of diabetic kidney disease. In the present study, urine was collected from DM and DKD patients with a duration more than 10 years and urinary exosomal miRNA profiling was conducted in urinary exosomes obtained from three patients with type 2 diabetes (DM) and three patients with type 2 diabetic kidney disease (DKD) using Exiqon's microRNA arrays. In total, the expression of 14 miRNAs (miR-4491, miR-2117, miR-4507, miR-5088-5P, miR-1587, miR-219a-3p, miR-5091, miR-498, miR-4687-3p, miR-516b-5p, miR-4534, miR-1275, miR-5007-3p, and miR-4516) was up-regulated (>2-fold) in DKD patients compared to healthy controls and DM patients. We used qRT-PCR based analysis of these 14 miRNAs in urinary exosomes from 14 DKD to 14 DM patients in confirmation cohort, among which seven miRNAs were consistent with the microarray results. The expressions of miR-4534 and miR-516b-5p correlated with trace proteinuria levels in the confirmation cohort. In conclusion, it has been confirmed that the expression of urinary exosomal miRNA in patients with type 2 diabetes DKD has changed. Mir-4534 might affect the FoxO signaling pathway by targeting BNIP3, and is expected to become a new biomarker for the progression of type 2 DKD disease, which will provide further research on the pathogenesis of DKD.

## Introduction

Diabetic kidney disease (DKD) is a common cause of end-stage renal disease (ESRD) worldwide, but the true incidence and prevalence of ESKD from diabetes is impossible to know because kidney biopsies [the gold standard for diagnosis of diabetic kidney disease (1)] are infrequently performed in diabetic patients with diabetic kidney disease (2). Meanwhile, there are some limitations of using albuminuria and glomerular filtration rate (eGFR) for diagnosing DKD. Some conditions, including exercise within 24 h, high-protein diet, infection, and fever, might result in falsely elevated albuminuria without any kidney damages (3). Moreover, these markers are usually a late sign of kidney damage. Recent studies suggested that urinary exosomal miRNAs, recognized a kind of potential non-invasive biomarker for early diagnosis and therapy of DKD, can help us understand the pathophysiological mechanisms leading to renal damage (4).

Exosome is a nanoscale vesicle with a size range of 30–100 nm. It is secreted from cell to extracellular space by exocytosis after fusion of multivesicular body (MVB) with plasma membrane (5). Exosomes, having crucial roles in cell-to-cell communication, through targeting their cargos such as miRNAs, mRNAs, DNAs, and proteins, all of which could influence on cellular pathways and mediate their physiological behaviors including cell proliferation, tumorigenesis, differentiation, and so on (6). It has been confirmed in DKD (7) that hyperglycemia causes dysfunction of endothelial and mesangial cells and changes in exosome components secreted by them, which is one of the important causes of glomerular fibrosis and podocyte injury. Generally considering, the urinary exosomes come from urogenital s system cells, as plasma exosomes could not pass through the glomerular filtration machinery (8). Exosomes secreted by renal cells along the nephron can be released into urine or enter target cells through several mechanisms so as to affect a series of pathphysiological processes of target cells such as transcription and translation by releasing proteins or miRNAs. The bilayer membrane structure of urinary exosomes isolates miRNA from RNase in urine, which can reduce its degradation, protect relatively intact kidney cell information, and provide a rich source of biological information for DKD diagnosis (9). Therefore, variations in urinary exosomes number, origin, or content may mirror the physiopathological state of the kidney (10). MiRNA is a mature epigenetic regulator and gene expression regulator. It usually silences the expression of the target gene by hybridizing with the target mRNA 3'UTR, resulting in translation inhibition or mRNA degradation (11). Barutta et al. (12) found that urinary exosomal miR-130a and miR-145 were significantly increased in DKD patients, while miR-155 and miR-424 were decreased. *In vitro* experiments have confirmed that in early animal models of experimental diabetic kidney disease, urinary exosomal miR-145 levels are elevated, parallel to miR-145 overexpression in the glomeruli. Mohan et al. (13) pointed out that the relative increase in miR-451-5p and miR-16 appears to have protective effects on renal fibrosis caused by diabetes after evaluating the tubulointerstitial fibrosis index and glomerulosclerosis index. MiR-451-5p may be a sensitive and accurate non-invasive indicator for the diagnosis of early DKD. At present, miRNA arrays have become one of the most widely used methods for screening biomarkers and exploring disease biological processes.

In this study, we identified characteristically altered miRNAs from urinary exosomes in patients with type 2 DKD. This dysregulation of unique urinary exosomal miRNA species is related to the extent of microalbuminuria. It is worth noting that the enriched expression of miRNA-4534, which is indirectly involved in FOXO signaling by targeting BNIP3, may represent a novel biomarker for disease progression and / or early treatment effects.

## Materials and Methods

### Patients and Urine Sample Processing

Statements to confirm that all methods were carried out in accordance with relevant guidelines and regulations (Declaration of Helsinki). All experimental protocols were approved by Ethics Committee of the First Affiliated Hospital of Zhengzhou University. Informed consent was obtained from all subjects or, if subjects are under 18, from a parent and/or legal guardian.

Inclusion criteria:

1. All selected diabetic patients met the WTO diagnostic criteria for diabetes: 1. typical diabetic symptoms (polydipsia, polydipsia, polyuria, and weight loss) and random blood glucose ≥ 11.1 mmol/L. 2. Fasting plasma glucose (FPG) ≥7.0 mmol/L; Fasting state refers to at least 8 h did not eat calories. Oral glucose tolerance test (OGTT) 2 h blood glucose ≥11.1 mmol/L. Take sugar water equivalent to 75 g of anhydrous glucose (venous blood was drawn from all patients). All patients with diabetes had a disease course of more than 10 years, no diabetic kidney disease (24 h urinary microalbuminuria <30 mg/24 h) and no retinopathy in the current and past medical history.
2. All the enrolled DKD patients met the above WTO diagnostic criteria for diabetes, and were associated with dominant proteinuria (24 h urinary microalbuminuria ≥300 mg/ 24 h) and retinopathy.

Exclusion criteria:

1. Patients with type 1 diabetic kidney disease and other special types of diabetic kidney disease;
2. Patients with various other glomerular diseases and renal interstitial diseases;
3. Patients with kidney stones and urinary tract infections;
4. Patients with autoimmune system diseases, malignant tumors, and blood system diseases;
5. Patients with severe chronic heart and lung disease, chronic liver, and kidney disease;
6. Patients with severe infectious diseases;
7. Application of glucocorticoids, immunosuppressants, or cytotoxic drugs, etc.

100 mL of first-morning urine was collected from all subjects in sterile containers. Urine samples were centrifuged at 3,000 g for 30 min at 4°C to separate cells from debris. 0.22 μm filter (or filter head) for filtering to remove bacteria, residual cells and debris. The pellet was stored at −80°C for subsequent applications. All the samples were processed within 1 h after collection. In order to gain an expression profile of urinary exosomal miRNAs that is specific to DKD, the microarray was used to identify the differentially expressed miRNAs in DM group (*n* = 3), and DKD group (*n* = 3) in the initial screening cohort. The rest subjects were divided into DM group (*n* =14) and DKD group (*n* = 14) for further analysis by qRT-PCR in confirmation cohort.

### Urinary Exosomes Isolation

Exosomes were isolated from 5 ml urine by using Exosome Isolation Kit (American System Biosciences) according to manufacturer's instructions. Morphological was performed by using Scanning Electron Microscopy (SEM) which further confirmed the authenticity of the exosomes. Briefly, the exosomes (isolated from the urine) were processed and visualized under “SEM” at 80000X magnification.

### Western Blotting

The protein extracts were separated using 10% SDS-PAGE and transferred onto a PVDF membrane (Millipore). The membranes were blocked with 5% evaporated skimmed milk in TBS (50 mmol/L Tris-HCl, pH 7.5, 150 mmol/L NaCl) containing 0.1% Tween-20 for 2 h and incubated overnight at 4°C with the appropriate primary Ab, followed by incubation with HRP-coupled secondary Ab for 1 h at room temperature. Furthermore, the protein bands were visualized on photographic film using ECL blotting detection reagents (P0018; Beyotime). The following primary antibodies were used for western blotting: anti-CD9, anti-CD63, and anti-TSG101 (Proteintech, America).

### miRNA Microarray Analysis

The microarray analysis for miRNA profiling was conducted by the Shanghai Kangcheng Technology using the miRCURY LNA Array system (Exiqon, Vedbaek, Denmark). Total RNA was extracted and purified using the mirVana miRNA Isolation Kit (Ambion, Austin, TX, USA) following the manufacturer's instructions. The miRCURY™ Hy3™/Hy5™ Power labeling kit (Exiqon, Vedbaek, Denmark) was used according to the manufacturer's guideline for miRNA labeling. After stopping the labeling procedure, the Hy3™-labeled samples were hybridized on the miRCURYTM LNA Array (v.19.0) (Exiqon) according to array manual. Following hybridization, the slides were achieved, washed several times using Wash buffer kit (Exiqon). Expressed data were normalized using the Median normalization. After normalization, significant differentially expressed miRNAs between two groups were identified through Fold change (>2) and *P*-value (<0.05).

### qRT-PCR Validation

Total RNA was extracted and purified using the mirVana miRNA Isolation Kit (Ambion, Austin, TX, USA) following the manufacturer's instructions. RNA was reversely transcribed to cDNA by using the TaqMan MicroRNA Reverse Transcription Kit with miRNA-specific stem-loop primers. The quantitative RT-PCR reaction was performed in 96-well plates on the ABI PRISM 7000HT termocycler by using Taqman Gene Expression assay according to the manufacturer instructions. The data were normalized based on the expression of the endogenous control miR-16 (14, 15).

### Data Analysis

Statistical analysis was performed by using SPSS 20.0 statistical software (SPSS, Inc.) and graphs were generated by using GraphPad Prism 8.0. Mann–Whitney *U*-test was used to compare miRNA expression levels between groups. Spearman's rank-order correlation was used to analyse the correlation between two parameters. The level of significance was set at ^*^P < 0.05. Error bar represent +/-SEM. The diagnostic performance of biomarkers was evaluated by calculating their sensitivity and specificity using ROC curves. To predict target genes and related pathways of deregulated miRNA, miRWalk (http://mirwalk.umm.uni-heidelberg.de/), TargetScan (http://www.targetscan.org/vert_72/), and miRDB (http://www.mirdb.org/) were used. Pathway enrichment of dysregulated miRNA was performed by DAVID (https://david.ncifcrf.gov/).

## Results

### Patients Characteristics

Clinical and laboratory characteristics of recruited subjects are shown in Tables 1, 2. DM group and DKD group patients were comparable in age, sex, diabetes duration, and BMI (*p* > 0.05). As expected, DKD group had worse renal function with higher micro-albuminuric (UALB) and eGFR than controls and DM group (*p* < 0.01). Furthermore, DKD patients were associated with diabetic retinopathy.

**Table 1:** Clinical and Laboratory parameters of controls, DM and DKD patients from the screening cohort.

	**DM**	**DKD**
*N*	3	3
Age (years)	59.67 ± 4.93	60 ± 12.16
Sex (female/male)	0/3	0/3
Diabetes duration (years)	15.33 ± 3.21	14 ± 10.39
BMI (kg/m^2^)	26.43 ± 1.82	24.03 ± 1.03
UALB (mg/24 h)	9.79 ± 2.42	1127.00 ± 1393.455*
eGFR (ml/min/1.73m^2^)	94.00 ± 14.47	85.39 ± 14.95*
Diabetic retinopathy	No	Yes*

*The data are expressed as the mean ± SEM, unless noted otherwise. BMI, Body Mass Index; UALB, micro-albuminuria; eGFR, glomerular filtration rate*.

* Significant differences against controls and DM group are indicated by *P < 0.05).

**Table 2:** Clinical and Laboratory parameters of DM and DKD patients from the confirmation cohort.

	**DM**	**DKD**
*N*	14	14
Age (years)	57.79 ± 8.51	55.5 ± 7.36
Sex (female/male)	7/7	7/7
Diabetes duration (years)	12.93 ± 3.58	11.36 ± 7.02
BMI (kg/m^2^)	25.73 ± 4.13	27.60 ± 7.62
UALB (mg/24 h)	12.85 ± 5.96	2394.90 ± 2887.70*
GFR (ml/min/1.73 m^2^)	96.66 ± 12.52	75.69 ± 33.82*
Diabetic retinopathy	No	Yes*

* Significant differences against DM group are indicated by *P < 0.05.

### Characteristics of Isolated Urinary Exosomes

**Figure 1:**
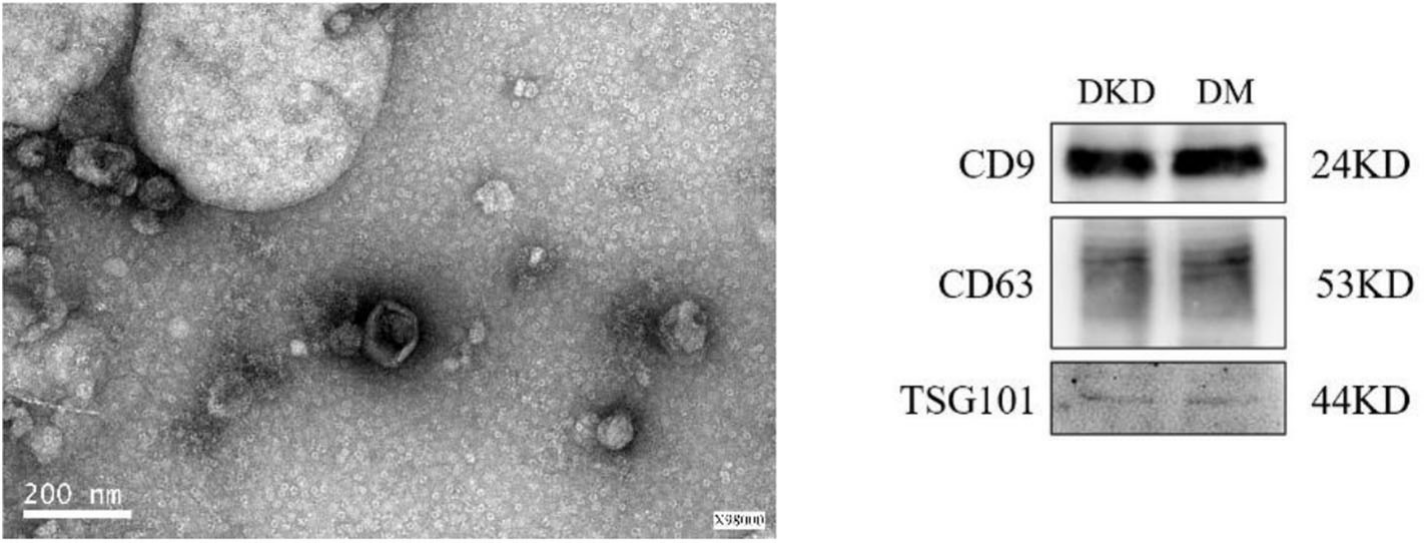
Exosomes under electron microscope; the characterization exosomes with related-markers.

The extracted microvesicles were cup-shaped, under 200 nm in diameter, and scattered as shown in Figure 1. The characteristic exosomes with relevant markers (CD9, CD63, and TSG101) can be detected in DKD and DM group.

### Differentially Regulated miRNA-Signature in Urinary Exosomal miRNA in the Screening Cohort

**Figure 2:**
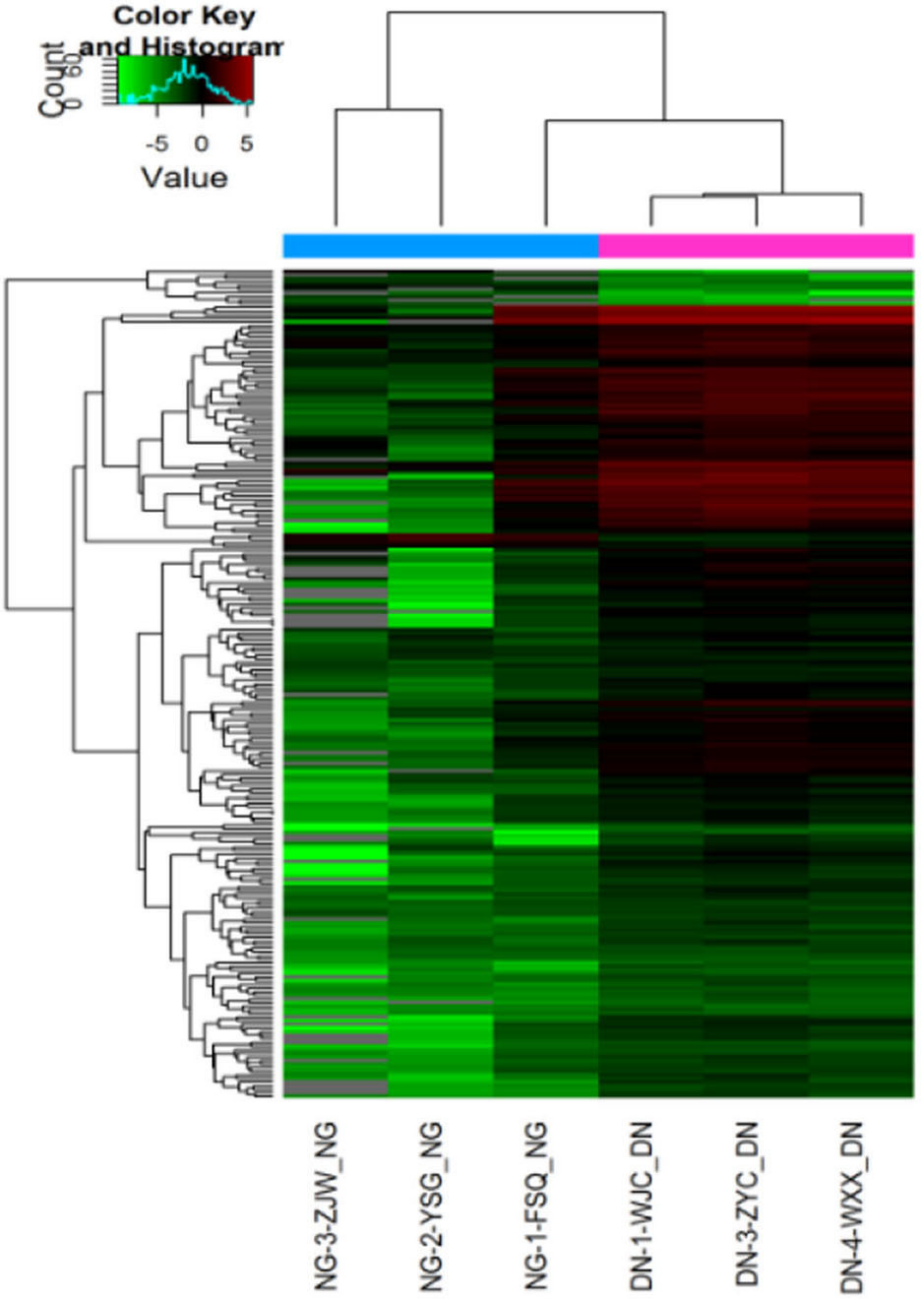
Heat map of expression levels of differentially expressed miRNAs in DKD patients in the screening cohort. Fold change of expression levels were normalized to the mean signal intensities of healthy controls. Red and green colors respectively represent the up- and down-regulation of fold change, as indicated by the linear scale bar.

**Figure 3:**
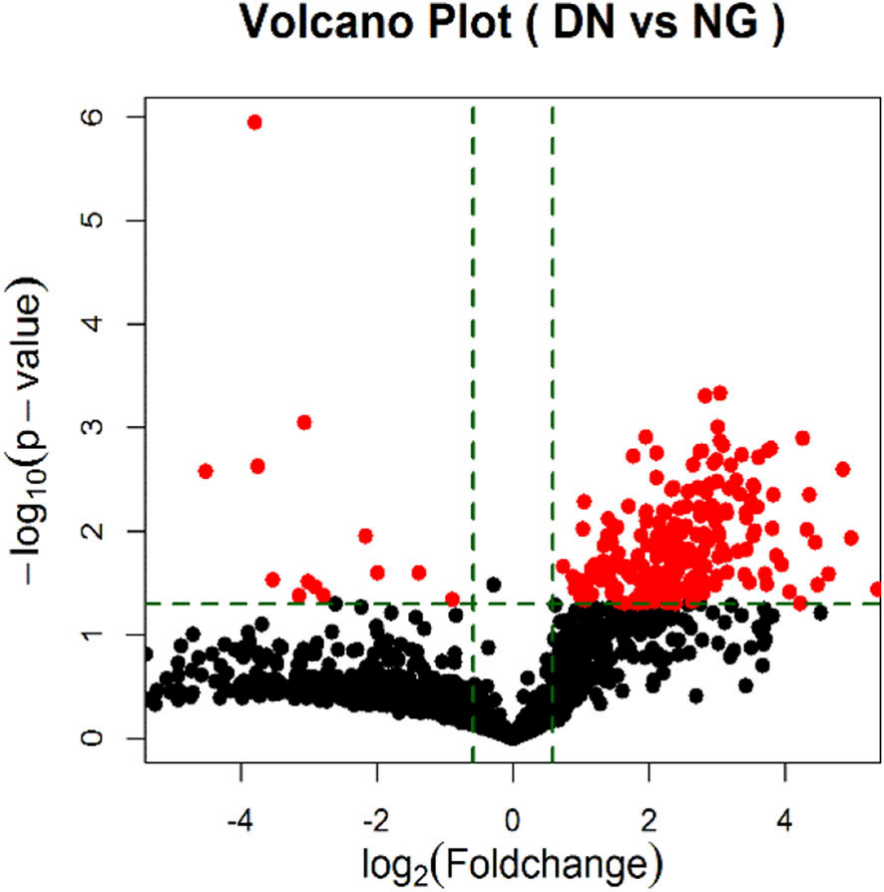
Volcano Plot of foldchange of differentially expressed miRNAs in DKD patients. Red and green colors represent fold change >2 and <2 (NG = DM patients; DN = type II diabetic kidney disease patients).

According to the results of urinary exosomal microarray, a total of 2,085 miRNA were detectable from DKD patients (Fold change > 5, *P* < 0.05, Figures 2, 3). 326 miRNAs showed significant upregulation in the exosomes of DKD group compared to DM group. Among these miRNAs, 212 are up-regulated and 14 are down-regulated. Of these, 14 miRNAs (miR-4491, miR-2117, miR-4507, miR-5088-5P, miR-1587, miR-219a-3p, miR-5091, miR-498, miR-4687-3p, miR-516b-5p, miR-4534, miR-1275, miR-5007-3p, and miR-4516) showed upregulation (Table 3). miR-4491 showed the strongest up-regulation (28-fold). The expression levels of mirna-2117, mirna-498, and mirna-4687-3p were the highest in the DKD group, and mirna-1275 followed. Studies have shown (16) that serum miR-4534 was up-regulated in patients with type 2 diabetes, and miR-1587 and miR-4516 may be respectively involved in the development of polycystic kidney disease (17) and hypertensive nephropathy (18).

**Table 3:** Different expression of the miRNAs detected according to the miRNA expression microarray.

	**Fold change**	**Expression**
miR-4491	28.603	2742.00*
miR-2117	4.243	11656.33*
miR-4507	13.020	2332.17*
miR-5088-5P	5.412	915.00*
miR-1587	11.501	1397.33*
miR-219a-3p	7.038	3696.17*
miR-5091	13.378	1354.50*
miR-498	9.680	12058.50*
miR-4687-3p	9.185	12285.50*
miR-516b-5p	5.192	1296.17*
miR-4534	6.863	1830.33*
miR-1275	8.991	6630.83*
miR-5007-3p	5.822	1513.33*
miR-4516	6.091	1370.00*

* Significant differences against DM group are indicated by *P < 0.05.

### qRT–PCR Confirmation of miRNAs Associated With DKD

**Figure 4:**
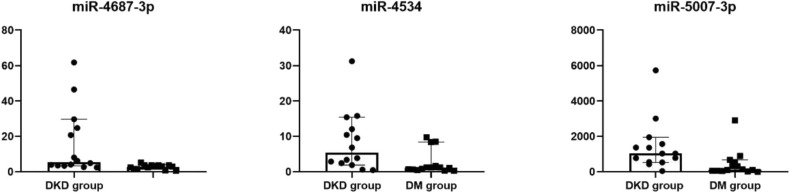
Dot plot of differentially expressed miRNAs in confirmation cohort. Differentially expressed in urinary exosomes in confirmation cohort, as indicated by Dot plot. The expression of miRNAs was higher in DKD group compared with DM group.

Fourteen miRNAs of urinary exosome microarray were verified by qRT-PCR in 14 diabetic patients and 14 diabetic kidney disease patients. We measured miRNA levels in urinary exosome. As shown in Table 4, Figure 4, the relative expression level of three miRNA speciesmiR-4687-3p, miR-4534, and miR-5007-3p exhibited increased in the DKD group compared with DM group (*p* < 0.05). However, the remaining nine miRNAs were not statistically different. The *P-*values was adjusted by the Benjamini and Hochberg method. The miR-5007-3p showed the strongest up-regulation of all miRNAs (*P* < 0.01).

**Table 4:** The relative expression level of urinary exosomal miRNAs in confirmation cohort.

	**DM**	**DKD**	***P*-value**	**FDR**
miR-4687-3p	3.09 (1.62–3.69)	5.55 (3.53–25.99)	0.002	0.028*
miR-4534	1.14 (0.62–3.37)	7.53 (2.31–12.92)	0.010	0.046*
miR-5007-3p	121.23 (55.34–582.25)	1033.67 (548.86–1669.30)	0.003	0.021*

*The data are expressed as geometric mean (25–75th percentile)*.

*FDR (adjust P-value): False discovery rate*.

* Significant differences against DM group are indicated by *P < 0.05. Mann–Whitney U-Test.

### Correlations Between miRNAs and Microalbuminuria

**Figure 5:**
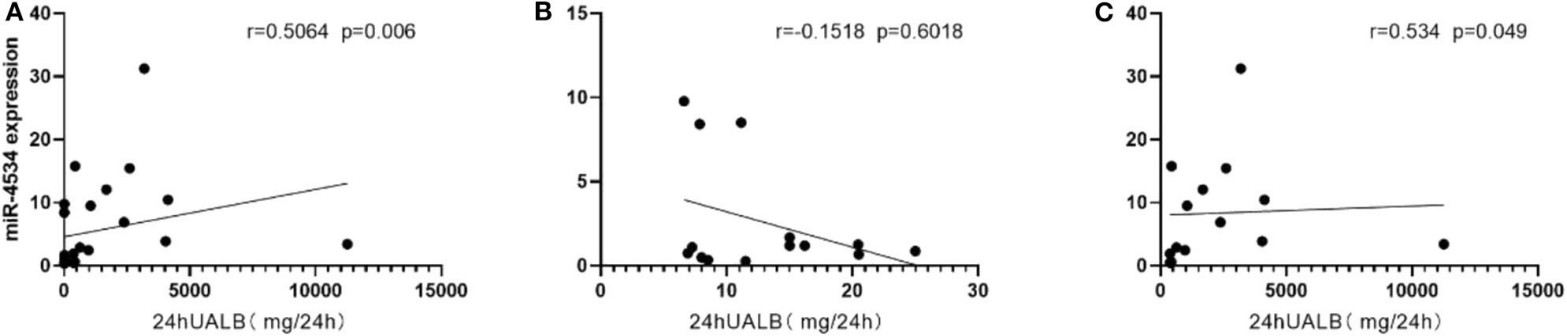
Scatter plot of correlations between miRNAs and microalbuminuria. **(A)** Correlation between miR-4534 expression in DM and DKD patients microalbuminuria in the confirmation cohort. **(B)** Correlation between miR-4534 expression in DM patients and microalbuminuria in the confirmation cohort. **(C)** Correlation between miR-4534 expression in DKD patients and microalbuminuria in the confirmation cohort. Data were compared by Spearman's correlation coefficient (*P* < 0.05).

Considering the situation, there is no significant difference in the diabetes duration between DM and DKD groups and correlation analyses revealed that up-regulated urinary exosomal miR-4534 (Figure 5A: *r* = 0.5604, *P* = 0.006) expression positively correlated with microalbuminuria (UALB). Furthermore, the correlation of miR-4534 and micro-albuminuria were stronger in DKD group (Figure 5C) whereas there was no correlation in the DM group (Figure 5B). No correlation was found with eGFR in confirmation cohort. Furthermore, miR-4687-3p and miR-5007-3P have no correlation with 24 h ALB and eGFR. ROC analysis revealed that miR-4534 had an area under the curve (AUC) of 0.786 (95% confidence interval, 0.607–0.965) (Table 5). Thus, we demonstrated that the quantification of miRNAs (particularly miR-4534) in urinary exosomes can be used as a primary or at least an auxiliary criterion for the diagnosis of DKD.

**Table 5:** Diagnostic value of miRNA in DM and DKD patients.

**miRNA**	**AUC**	***P***	**95% Confidence interval**		**Sensitivity%**	**Specificity%**
			**Upper limit**	**Lower limit**		
miR-4534	0.786	0.010	0.607	0.965	85.7	78.6

### Target Gene Prediction and Enrichment Pathway Analysis of Urinary Exosomal Up-Regulated miR-4534

**Figure 6:**
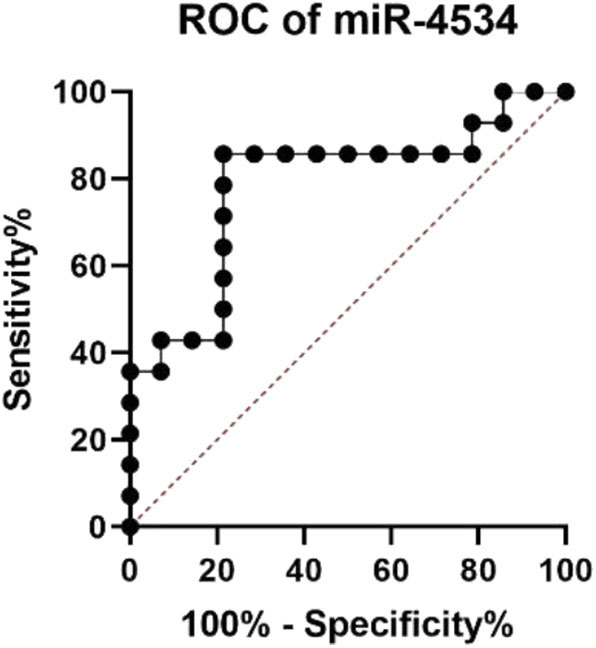
ROC analysis of miRNAs in DM and DKD patients.

**Figure 7:**
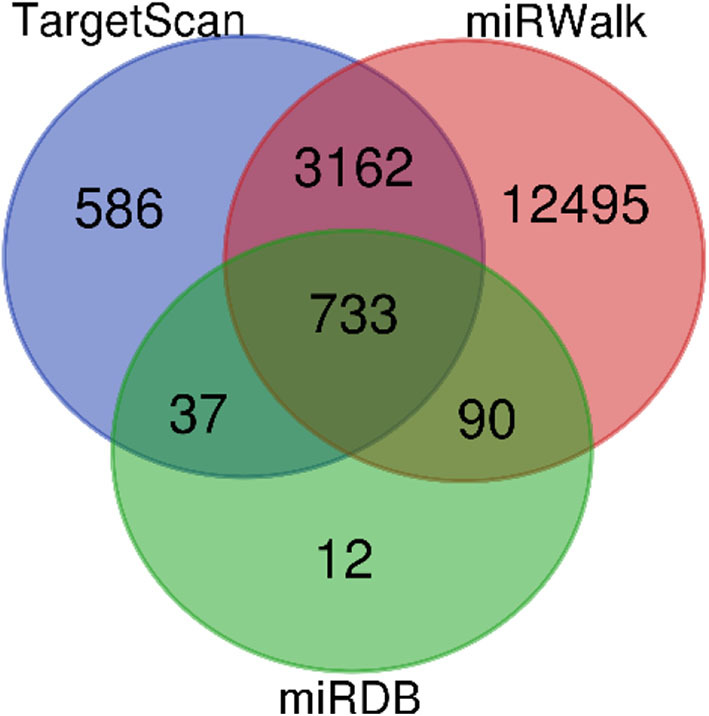
MiRNA-4534 gene prediction in Venn diagram. Four thousand five hundred and eighteen miRNA were detected by TaigetScan. One thousand six hundred and forty miRNA were detected by miRWalk. Eight hundred and seventy-two miRNAs were detected by miRDB. Seven hundred and thirty-three miRNA were simultaneously detected by three gene prediction websites.

**Figure 8:**
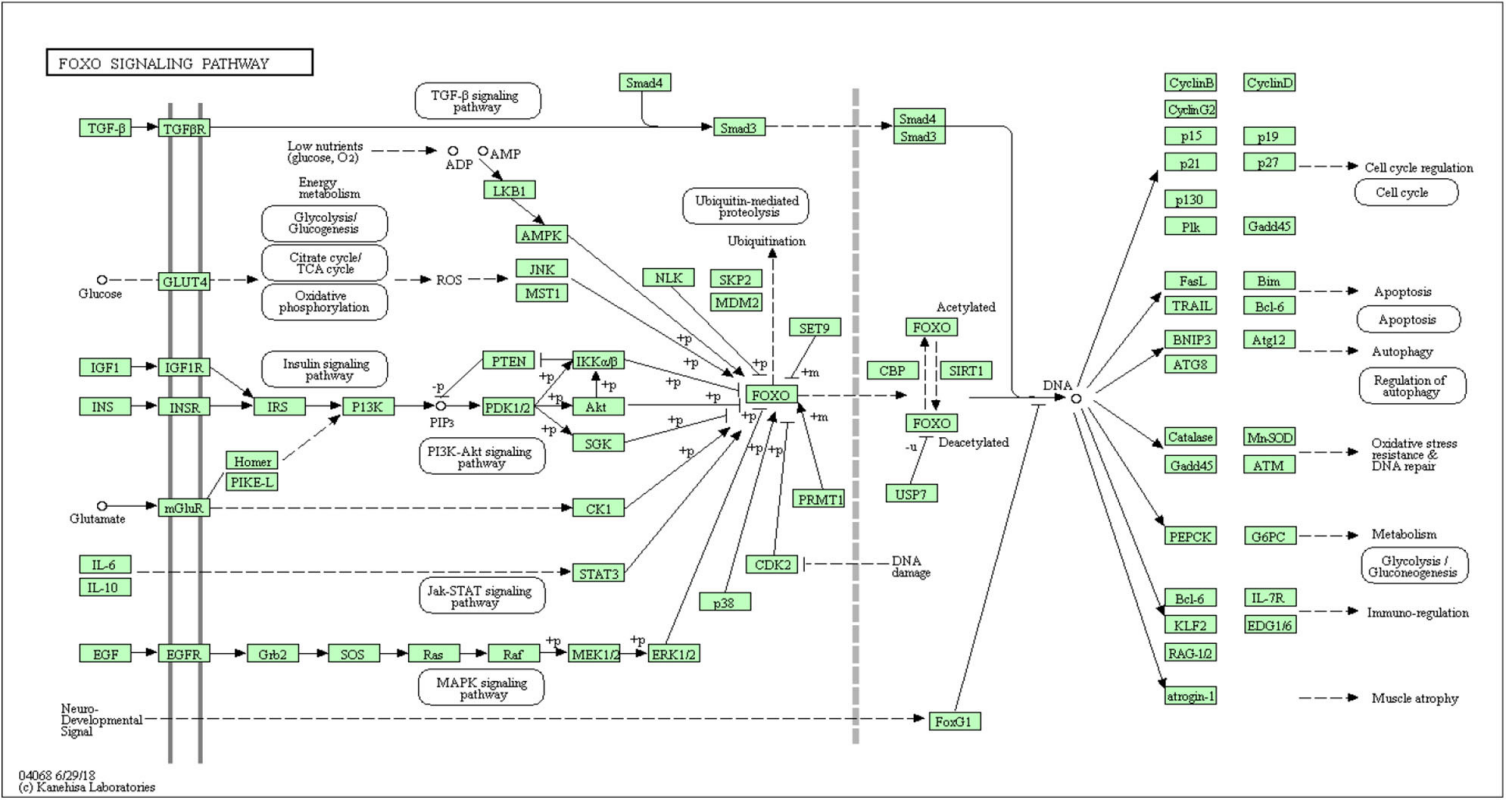
KEGG enrichment graph: miRNA-4534 is involved in the FOXO signaling pathway.

In all, it was predicted that miR-4534 probably involved in the regulation of genes and pathways, which provided a direction for further seeking the pathogenesis of DKD by using bioinformatics. Target genes of miR-4534 were predicted in the miRWalk, TargetScan, and miRDB databases respectively, and the intersections were analyzed on the DAVID website for enrichment pathway analysis. The results showed that there were 733 mRNAs involved in the intersection (Figure 6), and the enrichment pathway contained 10 pathways including glutamatergic synapses, axon guidance, neurotrophic regulatory pathways, transcriptional misregulation in cancer, and FOXO signal transduction. Based on the results of our research stated above, it was considered that miR-4534 may involve the progression of DKD by activating FOXO1/TXNIP-mediated oxidative stress pathway and aggravating podocyte injury (Table 6, Figures 7, 8).

**Table 6:** The KEGG analysis of urinary exosomal miR-4534.

**KEGG pathway**	**Count**	**Genes**
Glutamatergic synapse	12	GRM4, GLUL, GRIK3, DLG4, GNG13, PLA2G4F, GNG2, SLC38A1, GNG4, HOMER1, GRM1, SHANK2
Axon guidance	11	SEMA5A, PLXNC1, PLXNA3, SEMA3G, EFNB2, SEMA4B, EFNA5, UNC5D, NFATC3, SRGAP1, SRGAP2
Adrenergic signaling in cardiomyocytes	11	PPP2R1B, MYL2, ATP1B2, CACNG8, MAPK13, CAMK2D, CREB5, CACNG3, SCN7A, CALM1, CACNA2D4
Glioma	7	GRB2, ARAF, CAMK2D, IGF1, CDK6, PTEN, CALM1
Neurotrophin signaling pathway	10	RPS6KA6, IRAK3, MAPK13, GRB2, NFKBIE, CAMK2D, SORT1, TP73, ARHGDIB, CALM1
Transcriptional misregulation in cancer	12	CCNT2, PROM1, MAX, ETV7, KMT2A, REL, CCND2, LDB1, ETV1, IGF1, ETV6, PLAU
Dopaminergic synapse	10	PPP2R1B, DRD1, MAPK13, GNG13, CAMK2D, GNG2, CREB5, GNG4, CLOCK, CALM1
Cholinergic synapse	9	KCNQ5, KCNQ4, GNG13, CHRNB4, CAMK2D, GNG2, CREB5, CHRNB2, GNG4
FoxO signaling pathway	10	CCND2, MAPK13, GRB2, ARAF, SMAD3, IGF1, HOMER1, GRM1, PTEN, BCL2L11
GABAergic synapse	7	GABRG2, GABRE, GLUL, GNG13, GNG2, SLC38A1, GNG4

## Discussion

Exosomes contain proteins and genetic material (including miRNAs) derived from their parent cells and can potentially affect recipient cells. MicroRNAs (a class of non-coding RNAs) are strong regulators of gene expression. Exosome-related research is mostly focused on oncology, such as inflammation associated with cervical cancer (19) and the process of HIV replication and immune response (20). MiRNA has the potential to assist disease prognosis, diagnosis, and treatment effect prediction, as well as a target for disease treatment (21). The conjugation of miR-34a and GD2 antibody leads to an increase in the protein level of the tissue inhibitor metal titanase 2 precursor, which reduces angiogenesis in the tumor (22). However, research on endocrinology and nephrology is still in its infancy.

Some conventional approaches including next-generation sequencing, real-time polymerase chain reaction (PCR), northern blotting, and microarrays could be used for assessment of miRNAs expression, which can be used as a biomarker for risk assessment of stroke (23) and prognosis of tumor treatment (24). Surprisingly, exosomes play an indispensable role in regulating renal function (25). We did observe exosomes under the electron microscope (Figure 1). Exosomes secreted by kidney cells along the nephron are released into urine or into other kidney cells to release proteins or miRNAs. Those are involved in a series of pathophysiological processes (26). The urinary exosome microarray results showed that there were a large number of differentially expressed miRNAs in urine exosomes of patients with T2DM and DKD. We concluded that miR-4534 might be involved in the early formation of microalbuminuria. Delić et al. (27) analyzed miR-320c and miR-6068, the most strongly up-regulated miRNAs in urinary exosomes of DKD patients, and found that dysregulation of urinary exosomal miRNAs occurred in microalbuminuria DKD patients. Not in DM patients. Similar to what we found. After retrieval, miR-4534 is still a relatively novel RNA. Currently, there are no studies involving exosomes due to lack of sufficient understanding. In a study of differential expression of serum miRNAs in patients with type 2 diabetes (16), miR-4534 was found to be significantly increased in patients with T2DM. Although the study did not mention the cellular structures of urine and exosomes, given the exosomes' ability to transport “cargo” (28), there may be an intrinsic link between the two that we have not yet discovered.

Studies have shown (29) that miR-4534 is overexpressed in prostate cancer (PCA) and blocks cell proliferation, migration, induce G0/G1 cell cycle arrest and apoptosis, thus affecting tumor tissue growth, which may provide a therapeutic target and mechanism for the treatment of PCA in the future. In order to further explore the pathogenesis of DKD, our research group obtained a KEGG pathway map of miR-4534 through bioinformatics analysis (Figure 8), of which SIRT1/FOXO signaling pathway (30) is most closely related to DKD. It is well-known that studies on the pathogenesis of DKD are increasingly focused on inflammatory responses (31), and FOXO is one of the most well-known pathways. Our research group has been working on the pathogenesis of DKD for a long time and has investigated the role of FOXO1 variant in DKD for the first time (32). There is considerable evidence that FOXO1 is a regulator of autophagy (33), and a novel mechanism of vascular complications in diabetes has been found (34), namely, FOXO1 inhibits the expression of Atg14 to destroy autophag-lysosomal fusion, thereby mediating age-induced autophagy apoptosis in endothelial cells (ECs). On the basis of these findings, we may suggest that FOXO/BNIP3/Atg12 may be involved in the pathogenesis of DKD proteinuria.

Finally, with the in-depth research in the molecular field, the use of bioluminescent enzymes, fluorescent proteins, molecular beacons, and various nanoparticles to assess the function of exosomes and miRNAs is becoming more mature, looking forward to a major breakthrough in the field of DKD.

## Data Availability Statement

The original contributions presented in the study are publicly available. This data can be found here: https://www.ncbi.nlm.nih.gov/geo/query/acc.cgi?acc=GSE146337.

## Ethics Statement

The studies involving human participants were reviewed and approved by Ethics Committee of the First Affiliated Hospital of Zhengzhou University. The patients/participants provided their written informed consent to participate in this study.

## Author Contributions

YZ and GQ: conceived and designed the experiments. AS, FG, and YS: collected sample. YZ, AS, and NJ: performed the experiments. YZ and NJ: analyzed the data. HZ, JW, LW, and LF: helped perform the analysis with constructive discussions. YZ, AS, MP, XD, and XM: contributed reagents, materials and analysis tools. YZ: wrote the manuscript. All authors contributed to the article and approved the submitted version.

## Conflict of Interest

The authors declare that the research was conducted in the absence of any commercial or financial relationships that could be construed as a potential conflict of interest.

## Appendix

**Table:**

**Abbreviation List**	
**Abbreviation**	**Full names**
AKI	acute kidney injury
AUC	Area Under Curve
ATG	autophagy associated gene
AKT	Protein kinase B
BNIP3	bcl-2/adenovirus E1B 19kDa interacting protein 3
CKD	chronic kidney disease
DM	diabetes mellitus
DKD	diabetic kidney disease
FoxO	Forkhead box O
G	Gram
JAK	Janus kinase
miRNA	MicroRNA
Min	Minute
MBI	body mass index
UACR	Urinary Albumin /Creactinine Ratio
ESRD	end stage renal disease
GFR	glomerular filtration rate
MAPK	Mitogen-activated protein kinase
NF-κB	Nuclear Factor κB
Nm	Nanometer
PI3K	Phosphatidylinositol 3-kinase
PTEN	phosphatase and tensin homology deleted on chromosometen
ROC	receiver operating characteristic curve
RT-PCR	Real-time polymerase chain reaction
SOCS-1	suppressor of cytokine signaling-1
STAT	Signal transducer and activator of transcription,
TGF-β	transforming growth factor -β
UALB	urine microalbumin
UMOD	Uromodulin
**Primer sequence**	
**miRNA**	**Primer sequence**
hsa-miR-4491	AATGTGGACTGGTGTGACCAAA
hsa-miR-4507	CTGGGTTGGGCTGGGCTGGG
hsa-miR-1587	TTGGGCTGGGCTGGGTTGGG
hsa-miR-5091	ACGGAGACGACAAGACTGTGCTG
hsa-miR-4687-3p	TGGCTGTTGGAGGGGGCAGGC
hsa-miR-4534	TTTGGATGGAGGAGGGGTCT
hsa-miR-5007-3p	ATCATATGAACCAAACTCTAAT
hsa-miR-4516	TTGGGAGAAGGGTCGGGGC
hsa-miR-2117	TGTTCTCTTTGCCAAGGACAG
hsa-miR-5088-5p	CAGGGCTCAGGGATTGGATGGAGG
hsa-miR-219a-1-3p	AGAGTTGAGTCTGGACGTCCCG
hsa-miR-498	AAAGCACCTCCAGAGCTTGAAGC
hsa-miR-516b-5p	ATCTGGAGGTAAGAAGCACTTT
hsa-miR-1275	TTGTGGGGGAGAGGCTGTC
